# Emission from human skin in the sub THz frequency band

**DOI:** 10.1038/s41598-022-08432-5

**Published:** 2022-03-18

**Authors:** Noa Betzalel, Paul Ben Ishai, Alexander Puzenko, Yuri Feldman

**Affiliations:** 1 The Department of Applied Physics, The Hebrew University of Jerusalem, 9190401 Jerusalem, Israel; 2 The Department of Physics, Ariel University, 40700 Ariel, Israel

**Keywords:** Biophysics, Physics

## Abstract

Recently published Radiometric measurements of human subjects in the frequency range 480–700 GHz, demonstrate the emission of blackbody radiation from the body core, rather than the skin surface. We present a detailed electromagnetic simulation of the dermis and epidermis, taking into account the presence of the sweat duct. This complex structure can be considered as an electromagnetic bio-metamaterial, whereby the layered structure, along with the topology of the sweat duct, reveals a complex interference pattern in the skin. The model is capable of accurately representing the skin greyness factor as a function of frequency and this is confirmed by radiometry of living human skin.

## Introduction

Modern health care greatly benefits from non-invasive diagnostic techniques, especially if they are passive. Yet, there are surprisingly few methodologies for this. Beyond visual inspection, some work has been done on thermal imaging^1,2^, facial recognition^3^ and even using Doppler Radar^4^ to monitor heartbeat. However, the avenues for such monitoring are restricted by what can be emitted via human skin. Recently, Radiometry experiments on human subjects has revealed that in the sub-THz frequency band (480 GHz to 700 GHz) the emitted signal can reflect the level of stress experience by the person^5^. This work suggested the body core temperature as a source of blackbody radiation (T = 37 °C) that was modulated by its passage through the skin. To understand better the nature of transmission through the skin, one can use a simulation model. In this work, we perform analysis of electromagnetic (EM) response of the human skin in the frequency range of 500 GHz up to 700 GHz, by studying the simulated transmission coefficient, S_21_, in this frequency range. Using this approach, we clarify what part of the blackbody radiation passes the human skin to the outer world and what part of the skin, considered as a layered system with non-flat boundaries, is the dominant component in this mechanism. The simulation work is based on an EM human skin model that was developed in house^6,7^. The new model, optimized for the current analysis, contains the two sections of the sweat duct—the upper epidermal coiled outlet duct and the dermal duct outlet, which was previously ignored.

In the interaction of microwave radiation and human beings, the skin is traditionally considered as just an absorbing stratum sponge filled with water. In 2008 we demonstrated that such a view is inconsistent for the sub-THz band^8^. Human sweat gland ducts are coiled in the epidermis and, given their geometry, dimensions and electrical properties, could be regarded as EM entities^9,10^. We demonstrated that the sweat duct could have EM behaviours reminiscent of helical antennas. Experimental evidence was presented that the reflectance of the human skin in the sub-THz region strongly depends on the level of activity of the perspiration system^6,7,11^. Additionally, it correlates with physiological stress as manifested, e.g., by the Galvanic Skin Response (GSR), pulse rate and the systolic blood pressure^12,13^.

Recently, we modelled the human skin’s reflectance to an impinging sub-THz field using our model, based on the dimensions and dielectric parameters of the skin layers and structures like the sweat duct. Based on this model we have extracted the most suitable *ac* conductivity values of the sweat duct by fitting our simulation results to experimental values of reflection coefficient from human skin. An interesting outcome of this work is the recognition that future 5G transmissions will lead to standing wave absorptions in the skin, where the presence of the sweat duct will play a dominant role^6,7,14^. In the most recent work, a radiometric study of human emissivity around 500 GHz and 507 GHz was conducted on 32 volunteers. The experimental setup was based around a Superconducting Integrated Receiver (SIR)^15^. The SIR was used to measure the brightness temperature of subject’s skin at 500 and 507 GHz for the entire subject pool. Additionally, spectral measurements in the range 480–700 GHz were carried out on 3 subjects. Tuning the SIR for each frequency point is a time-consuming process. The results demonstrated the first evidence that human stress directly effects the emission of skin. In addition, the experiment suggested that the source of the emission was in fact blackbody radiation from the body core rather than the skin surface. To better understand this statement, one must investigate just how radiation in this frequency range passes through the skin and this can only be done by simulation. In the following sections we provide a brief scientific background of the structure of human skin. The human skin model is described and finally the simulation results, discussion and conclusions are presented.

## Scientific background

### Human skin anatomy

**Figure 1:**
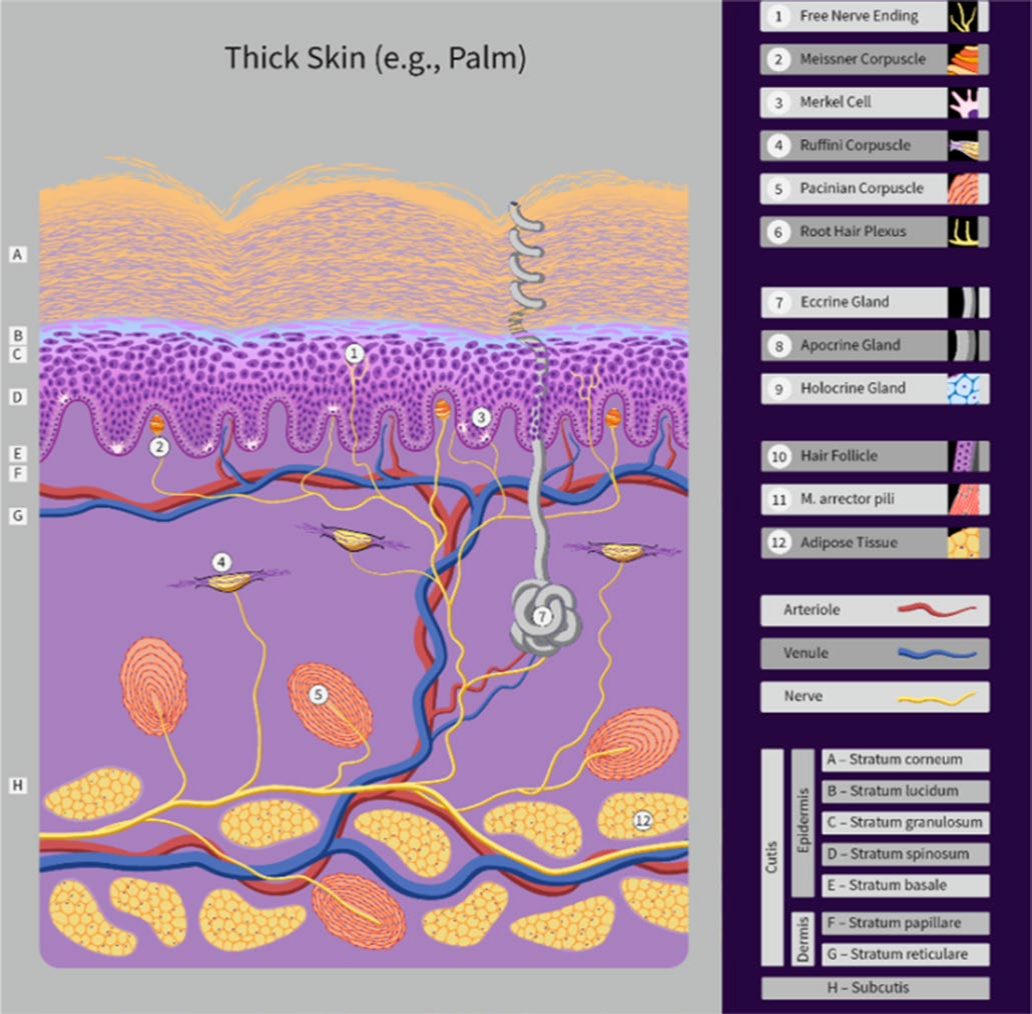
A schematic of human skin showing the main human skin layers and coiled sweat duct. (Modified from the original under a CC BY SA license, https://creativecommons.org/licenses/by-sa/4.0/. Copyright Guido Hegasy, https://www.hegasy.de/).

Human skin consists of three main layers: epidermis, dermis; and subcutaneous fatty layer. The outermost two layers, Epidermis and Dermis, are illustrated in Fig. 1, for the relatively thick skin of the hand.

**Figure 2:**
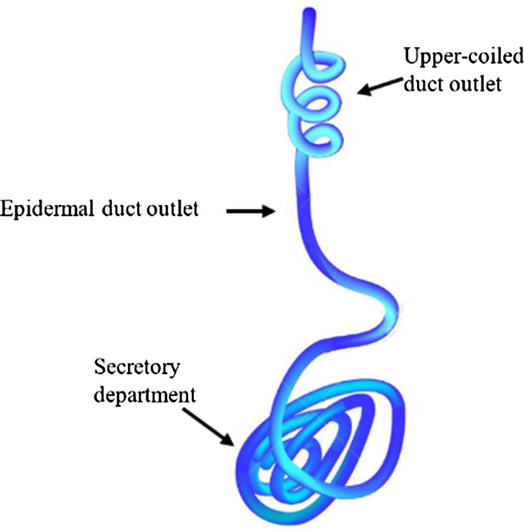
Schematics of eccrine sweat gland.

Our skin contains 2–5 million eccrine sweat gland ducts^16^. Each of which consist of three main parts: the secretory department, the dermal duct outlet and the upper-coiled outlet duct (see Fig. 2). The eccrine sweat glands are located at the bottom of the dermis, and are deployed throughout the skin. Optical Coherence Tomography (OCT) imaging of the human palm has revealed that the sweat duct in human skin is coiled in the epidermis layer, with a mean diameter of about 90 μm^17^ and a mean thickness of the epidermis of 270 μm^17^ (measured on a sample size of 32 subject).

**Figure 3:**
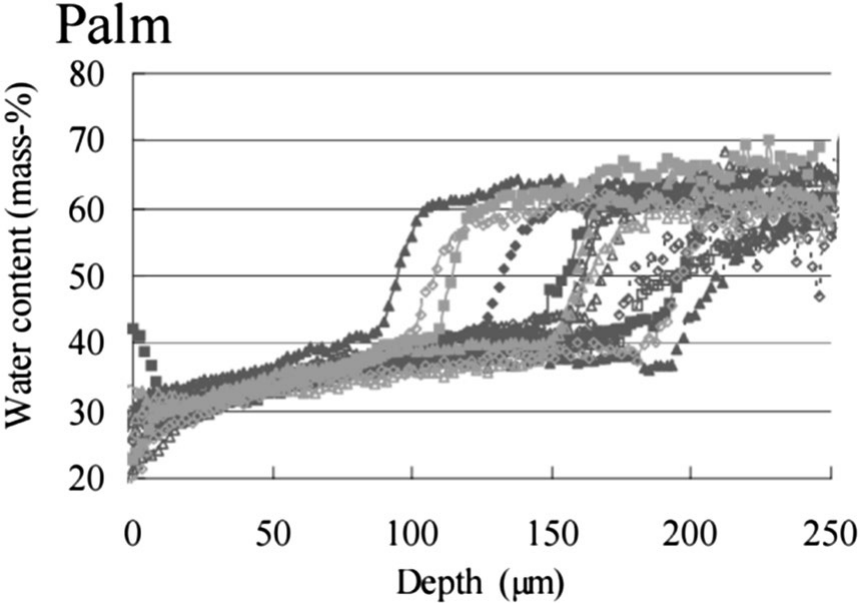
The epidermis water concentration profile as a function of the palm skin's depth measured in the palm of the hand of 15 subjects. The sharp rise in water content from approximately 40% to 65% mass represents the boundary between the epidermis and the dermis of the subject. The measurements were made in vivo, where Raman spectra were obtained at different depths below the skin surface using a confocal Raman spectrometer. The average depth of the epidermis was 173 μm with a standard deviation of 37 μm. (Reproduced with permission^21^. Published in Acta Dermato-Venereologica for the Society for Publication of Acta Dermato-Venereologica. All rights reserved).

The epidermis can be divided into sublayers. This depends on different areas of the skin, but is usually between 4 to 5 in number^18^. For the palm, 5 sublayers are considered. In general these layers consist of of sheets of keratinocytes, which constitute 90% of the cells found in skin, and these are nourished by the diffusion of intercellular fluids, from the dermal vasculature^19^. Keratinocytes undergo *‘keratinocytes migration’*, in which they migrate apically from the deep-most sublayer, the basal layer, and through the granular layers^20^. An accumulation of keratin cells ensues, which then undergoes terminal differentiation to generate the surface layer of cells, the Stratum Corneum (SC). Thus, the live keratinocytes cells’ concentration decreases as we move progressively towards the skin surface, leading to a water gradient throughout the epidermis layer. Figure 3 shows the epidermis water concentration profile as a function of the palm skin's depth, measured in the palms of the hand of 15 subjects. The measurements were made in vivo, here Raman spectra were obtained at different depths below the skin surface using a confocal Raman Spectrometer. The average depth of the epidermis was 173 μm with a standard deviation of 37 μm. Reproduced from Egawa et al.^21^; Copyright 2007, Advanced in Dermatology and Venereology).

It was shown that the helical structure of human eccrine sweat ducts, together with the dielectric properties of the human skin, has electromagnetic-properties that resemble to those of passive low Q-factor helical antennas^6–9,22,23^. The resonance frequency of such kind of antenna can be estimated using Eq. (1)^6^.

1 $$f = \frac{{c_{0} }}{{2\pi R \cdot \sqrt {\varepsilon_{eff} } }} \approx 530\;{\text{GHz}},$$

where $c_{0}$ is the velocity of light in free space, $R$ is the radius of the coiled duct, and $\varepsilon_{eff}$ is the effective dielectric permittivity of the dermis, calculated using a mixture formula as follows^22^:

2 $$\varepsilon_{layer} = \varepsilon_{bm} \cdot \frac{{\left( {2\varepsilon_{bm} + \varepsilon_{w} } \right) + 2\phi \left( {\varepsilon_{bm} - \varepsilon_{w} } \right)}}{{\left( {2\varepsilon_{bm} + \varepsilon_{w} } \right) - \phi \left( {\varepsilon_{bm} - \varepsilon_{w} } \right)}},$$

where $\varepsilon_{bm}$ is the dielectric permittivity of the dry biological structural components, and is estimated to be approximately 2.2^24^, $\varepsilon_{w}$ is the dielectric permittivity of water and $\phi$ is the volume fraction of the water component^22,24^. The details of the mixture formula application are specified in^6^. In Ref.^14^ the natural distribution in both individual coil diameter and water content was shown to lead to an uncertainty of 10% in the value of Eq. (1).

### Human skin black body radiation in sub-mm range

**Figure 4:**
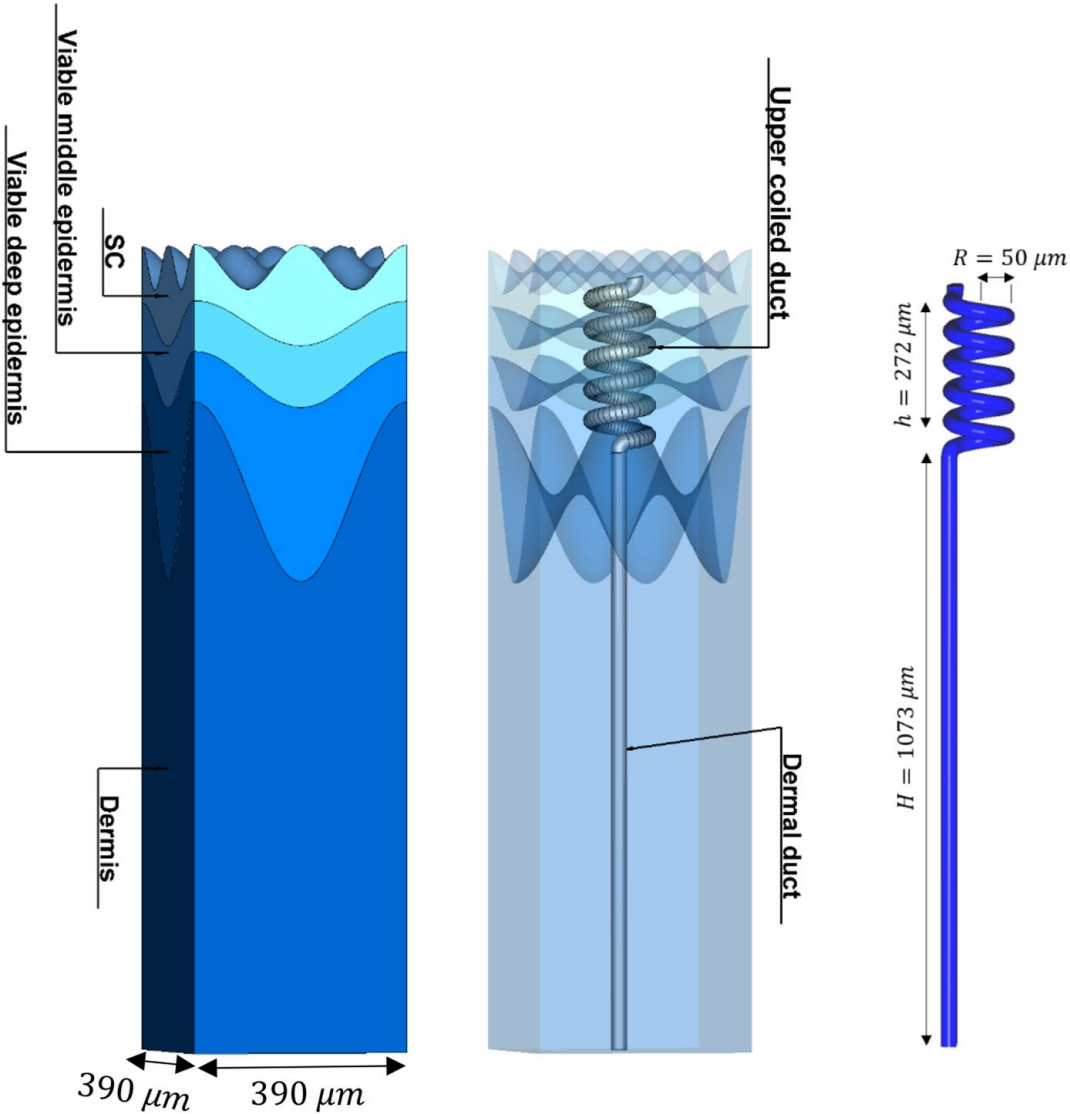
Electromagnetic thick human skin model designed and simulated using CST STUDIO SUITE, MICROWAVE STUDIO^30^. On the left—the whole model from an external view. On the middle—and interior view. As can be seen the coiled duct is fully embedded in the epidermis layer and the dermal duct is fully embedded in the dermis layer. On the right—the sizes of the duct which was used in the simulations. These values were based on the dimension of the layers and the sweat duct obtained in ref 17. The unit cell dimensions are 390 μm × 390 μm. This figure was generated using CST STUDIO SUITE v2021, https://www.3ds.com/products-services/simulia/products/cst-studio-suite/.

The thermal signature of a human being is a familiar image today^25^. Indeed, the human skin temperature of approximately 32 °C leads to an equivalent blackbody spectra in the Infrared^26^. The brightness temperature (or $T_{B}$) is the temperature of a blackbody in thermal equilibrium with its surroundings, in order to duplicate the observed intensity of a grey body object at a frequency *f*^26^ This concept is used in radio astronomy, planetary science and materials science^27^. The spectral radiance of a blackbody at temperature *T* and frequency *f*, according to Planck’s law, is given by:

3 $$B\left( {T,f} \right) = \frac{{2hf^{3} }}{{c^{2} }}\left( {e^{{\frac{hf}{{k_{B} T}}}} - 1} \right)^{ - 1} \;\left[ {\frac{{\text{W}}}{{{\text{m}}^{2} {\text{Hz}\Omega }}}} \right]$$

where *h* = 6.626 × 10^−34^ [Js] is the Planck constant, *c* = 3 × 10^8^ [m/s] is the speed of light and k_B_ = 1.38 × 10^−23^ [J/K] is the Boltzmann constant, and it is measured in terms of the power emitted per unit area of the body, per unit solid angle, per unit frequency. Although radiating in all frequencies, the main frequency of emission is defined by the temperature of the body and shifts as the temperature increases. At room temperature most of the emission is in the infra-red region of the EM spectrum (30 THz-450 THz). Actual blackbodies do not exist in nature, and real materials emit radiation only at a fraction of an ideal blackbody’s radiation. This fraction is described by a parameter called emissivity, which is equal to 1.0 for a perfect blackbody at a constant temperature, and lower than 1 for an actual physical body. A source with lower emissivity regardless of frequency, such as the human body at 37 °C, is referred to as a gray body^28,29^. As the human sweat duct can be considered as an EM entity, one could expect an influence of it on the emission of a blackbody (or a gray body) signal in the mentioned frequency band, because they are buried in the dermis and in contact with capillary blood system at 37 °C. In previous works, we have shown that the *ac* conductivity of the duct’s aqueous interior plays a major role in the value of the reflection coefficient^12–14^. Therefore, the question begs; Does the *ac* conductivity in the duct play a similar role in the emissivity? However, at the characteristic frequency of the duct, predicted by its geometry (see right hand side of Fig. 4), there would be a heighten absorption of the blackbody signal. This dependence on the spiral geometry of the duct is reminiscent of the absorption characteristic of a helical antenna, almost like a low-Q resonance, narrow band notch filter, as was shown by our group in^6–11,22,23^. In our recent study, we present results that demonstrate that human blackbody is having also a marked contribution in the sub-mm region, previously not noticed. Furthermore, this contribution is sensitive to the physiological state of the subject, opening a possible remote diagnostic channel^5^.

## Methods

### Human skin model

#### EM simulations

The EM human skin model is a 3-dimentional stratified structure of the main two layers: dermis and epidermis, where the last is further divided into three sub-layers. In total, the skin model is consist of four layers of different tissues. It is also includes two out of three parts of the sweat duct: the dermal duct and the upper-coiled duct. The skin model configurations are shown in Fig. 4 and the dielectric properties applied to the human skin model are presented in Table 1. The monotonically increasing water content in the epidermis, evident in Fig. 3, leads to a continual change in the effective dielectric permittivity, as defined by Eq. (2). To simplify the simulation and reduce computational load, this layer was approximated by dividing it into two sublayers, based on the average of the water content. In detail, the skin model layers, according to our internal notations, are:

- 1. *Epidermis* (upper most main layer):
  - *SC*: the outermost layer of the skin, consisting of dead keratinized cells (outermost sub-layer of the epidermis).
  - *Viable middle epidermis*: (middle sub-layer of the epidermis).
  - *Viable deep epidermis*: (deep most sub-layer of the epidermis).
- 2. *Dermis*: the outermost perfused skin layer (the middle main layer).
- 3. *Sweat duct*:
  - *Dermal duct*: straight duct outlet (embedded in the dermis layer).
  - *Upper coiled-duct*: epidermal spiral outlet duct (embedded in the epidermis layer).

**Figure 5:**
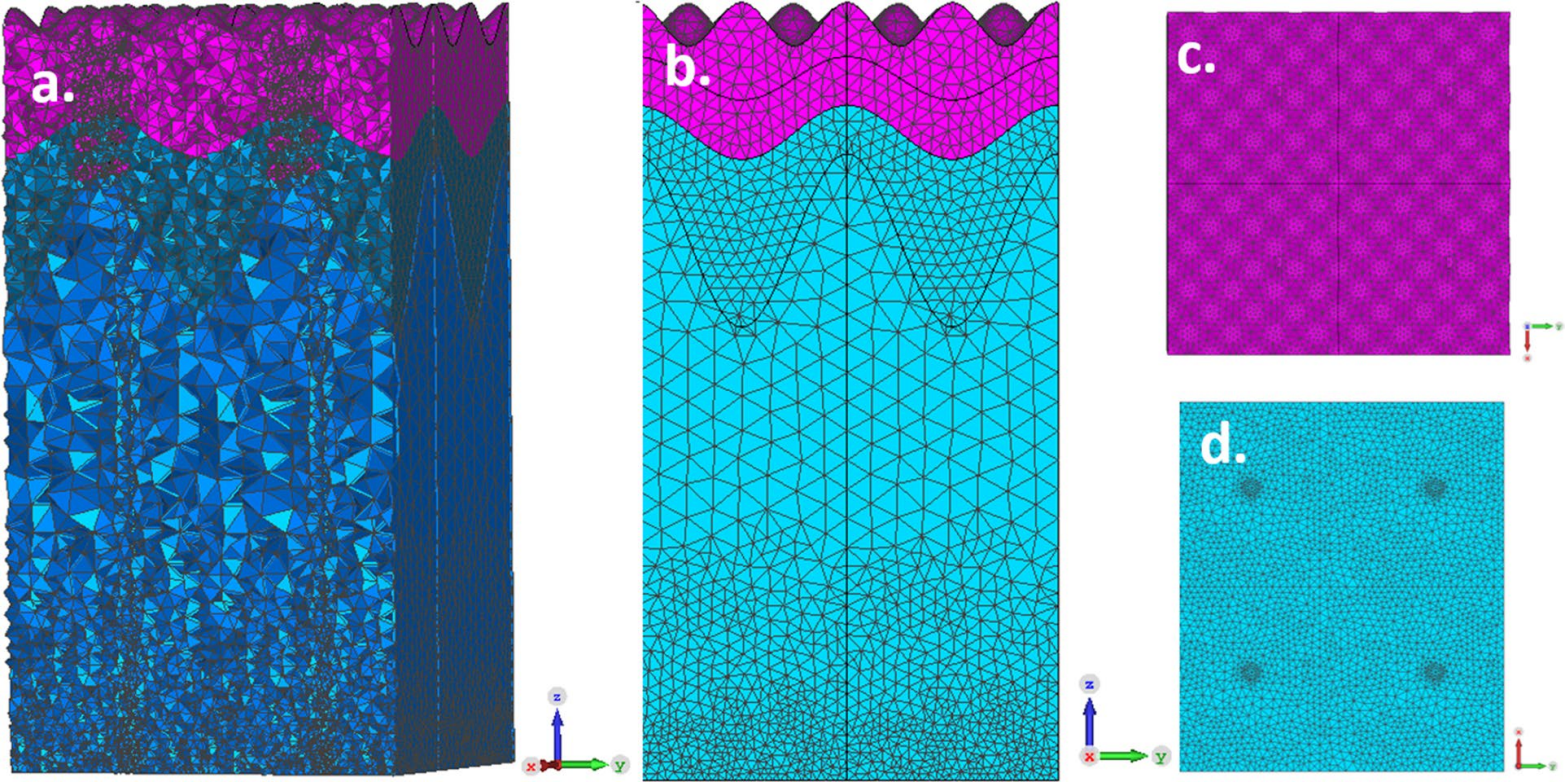
Mesh view of the human skin model. (**a**) a 3D view of the zy-plane, (**b**) a 2D view of the zy-plan. The adaptive mesh id clearly shown, as different areas of the model have different division of tetrahedral mesh cells, (**c**) top view. A more fine division to mesh cells is taking place due to the curved sinusoidal nature of this surface. (**d**) Bottom view. Here, as well, a finer mesh division is taking place to overcome the sharp edges of this surface. This figure was generated using CST STUDIO SUITE v2021^30^, https://www.3ds.com/products-services/simulia/products/cst-studio-suite/.

The CST STUDIO SUITE software with the MW MODULE^30^ was used (Release Version 2021.05—Jun 28, 2021) for the development of the current model. The Frequency-Domain solver was preferred over the Time-Domain solver for a number of reasons; especially its ability to solve high order elements in a curved mesh, its improved topology change and its detection of mesh move. The model was divided into over 200,000 tetrahedral mesh cells, in an adaptive manner. It means that the coiled portion of the sweat duct was meshed in a finer manner, due to its curvature, as well as the surfaces of the model. Figure 5 demonstrates the adaptive mesh method implemented to our model.

**Table 1:** Dielectric properties applied to the human skin model.

Skin component		Relative permittivity	AC Conductivity $\left( \frac{S}{m} \right)$
*Epidermis*	SC	2.76	0.001
	Viable middle epidermis	3.29	0.5
	Viable deep epidermis	3.8	1
*Dermis*		3.9	30
*Sweat Duct*	Upper coiled-duct	4	100–4000
	Dermal duct		

The values of permittivity were taken from Ref.^6^. The *ac* conductivity range of the sweat duct was taken from Ref.^14^.

### Computational method

**Figure 6:**
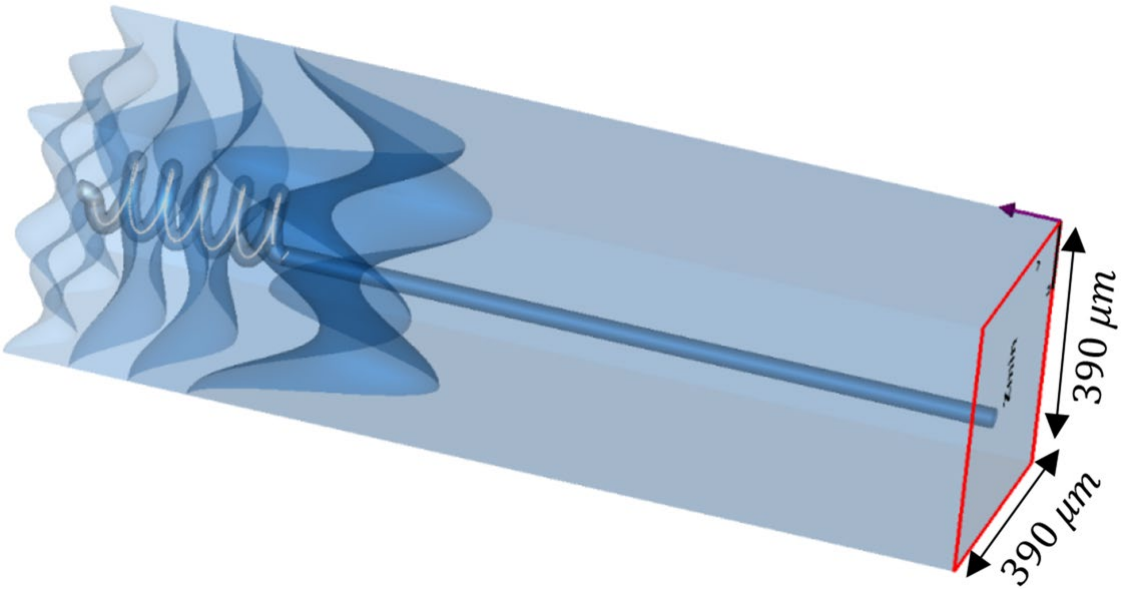
Electromagnetic excitation signal applied to the Z_min_ port, using 18 modes of Floquet. This figure was generated using CST STUDIO SUITE v2021^30^, https://www.3ds.com/products-services/simulia/products/cst-studio-suite/.

The accuracy of any simulation greatly depends on the boundary conditions as well as on the computational power available. In order to reduce the computational effort and remove boundary effects, a unit cell boundary condition was applied to the model, so allowing the application of Floquet theorem, which imitate a realistic EM source when being used with a large number of modes. Therefore, for a realistic signal input to the duct was, the first 18 modes of Floquet were applied to the model from its inner side. Namely, $Z_{min}$ port plane. The two fundamental Floquet mode TE(0,0), which is a linearly polarized plane wave, was selected to serve as the perpendicular excitation (see Fig. 6). The emphasis of this research was on the electrical field and current density distributions throughout the model. These distributions reveal the EM behavior of human skin in the sub-mm wave range, and gives a novel insight on the EM mechanism of human skin irradiation in this range.

In our previous work, we developed a method which demonstrate the link between the ac conductivity of the coiled sweat duct and the modulated EM skin response of the subjects to an external stimuli that would trigger perspiration14,11.

## Results and discussion

**Figure 7:**
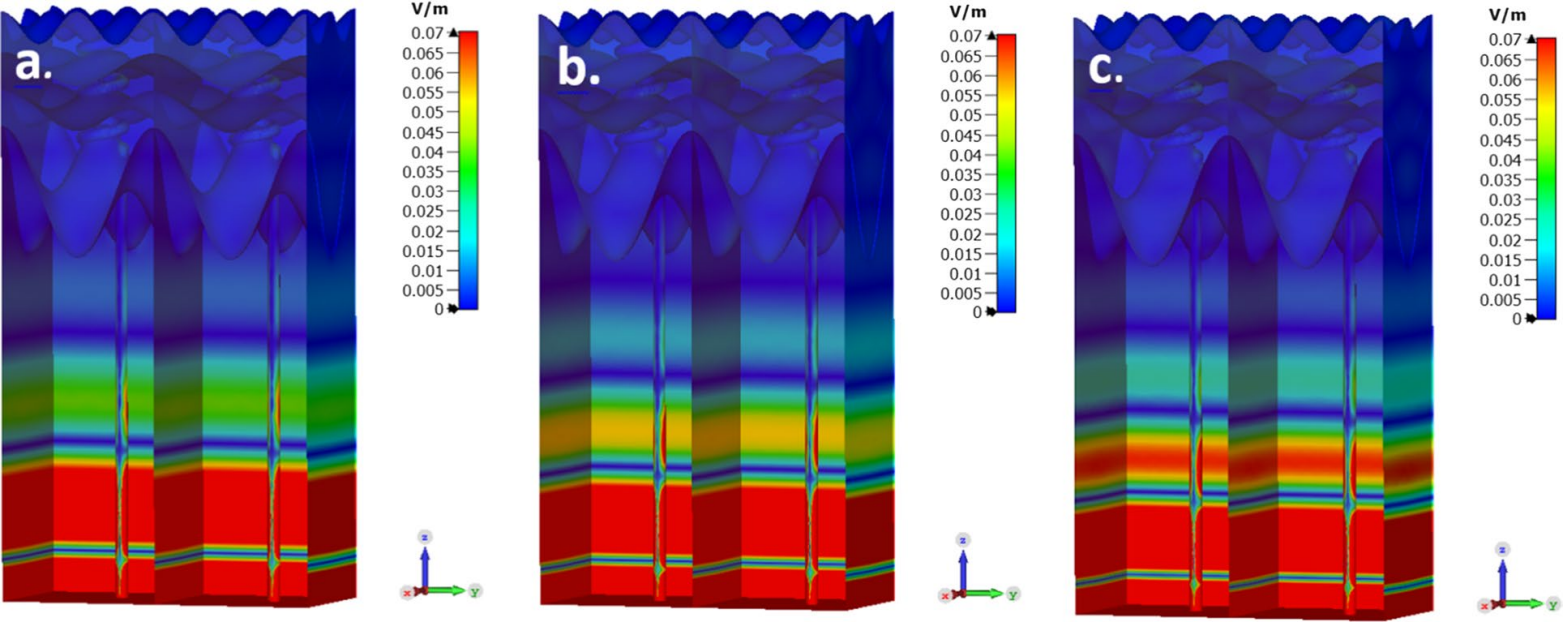
Electrical field distribution simulated for three of the nine different frequencies, which were simulated in the range of 500 GHz up to 700 GHz, with a constant frequency interval of 25 GHz. (**a**) 500 GHz, (**b**) 600 GHz and (**c**) 700 GHz. The duct conductivity is 1,000 S/m and power average of 5 nW. The phase for all frequencies was fixed to 145°. This figure was generated using CST STUDIO SUITE v2021^30^, https://www.3ds.com/products-services/simulia/products/cst-studio-suite/^30^.

Using EM simulations, the electrical field was simulated for nine different frequencies in the range of 500 GHz up to 700 GHz with a constant frequency interval of 25 GHz (500, 525, 550, 575, 600, 625, 650, 675 and 700 GHz). For each frequency, the field distribution was recorded for the same arbitrary fixed phase value of 145°. Figure 7 demonstrate the results of three of the nine frequencies which were simulated (500, 600 and 700 GHz) for an absolute distribution (The following results are shown for all nine frequencies in the figure S1 of the supplementary). All simulations were made for a sweat duct conductivity of 1,000 S/m and a plane wave source of average 5 nW (approximately 0.5 V/m).

**Figure 8:**
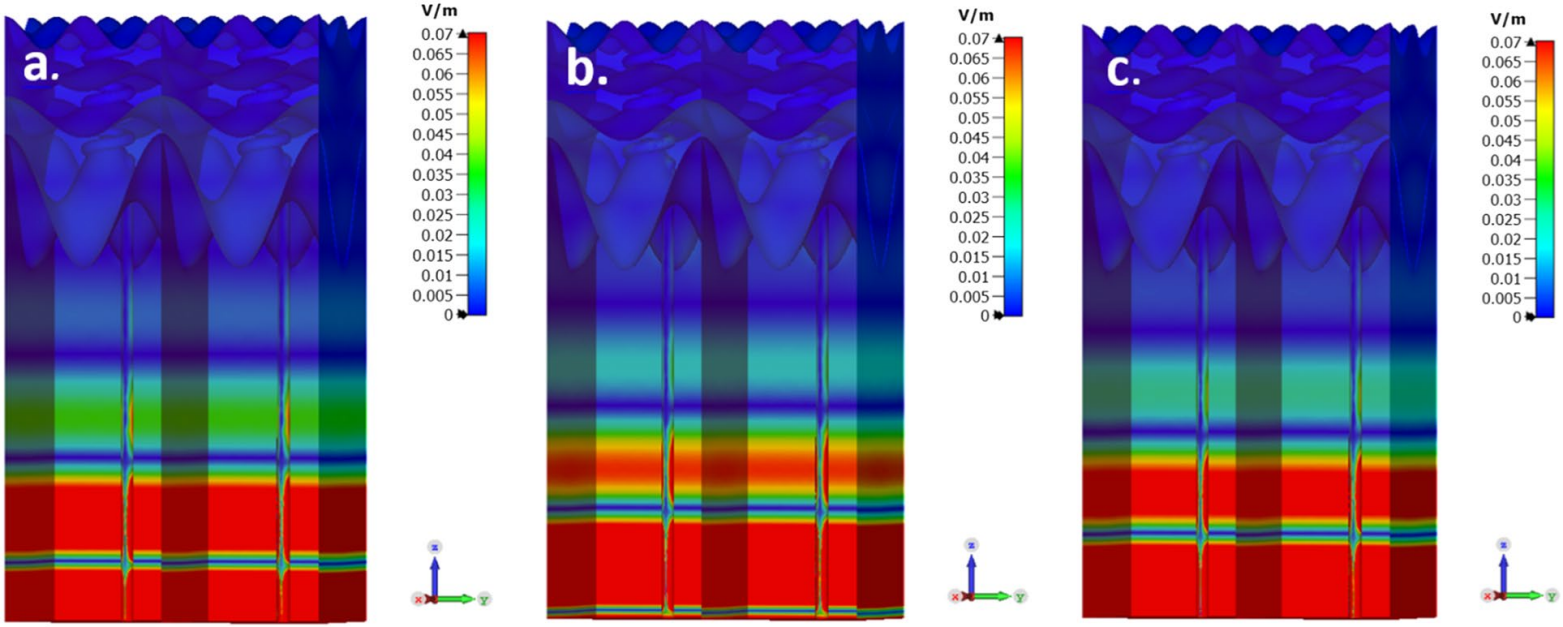
Fixed frequency of 550 GHz and different phases (**a**) 0, (**c**) 90° and (**f**) 225°) out of the nine which were simulated. In the scale-bar, all values above 0.07 V/m are marked in Red color. This figure was generated using CST STUDIO SUITE v2021^30^, https://www.3ds.com/products-services/simulia/products/cst-studio-suite/.

The simulated electric field shows the dermal duct and the bottom area of the dermis to be the skin components in which the field is mostly concentrated, even to the top of the duct. The simulation of the electric field reveals that is structured inside the layers, in particular, there is an interference pattern of standing waves. In Fig. 7 the red areas indicate field strengths above 0.07 V/m (the maximum field strength is 0.45 V/m at the duct bottom). The decay of the field envelop is exponential, as expected, and can be clearly seen in Fig. 10. In Fig. 7 the dependence on frequency of the standing wave structure, a clear etalon effect is clearly seen. In Fig. 8, the simulation is for a single frequency—550 GHz-but with different initial phases, representing the passage of the plain wave through the strata (The following results are shown for all nine phases, which were simulated, in the Fig. S2 of the supplementary).

**Figure 9:**
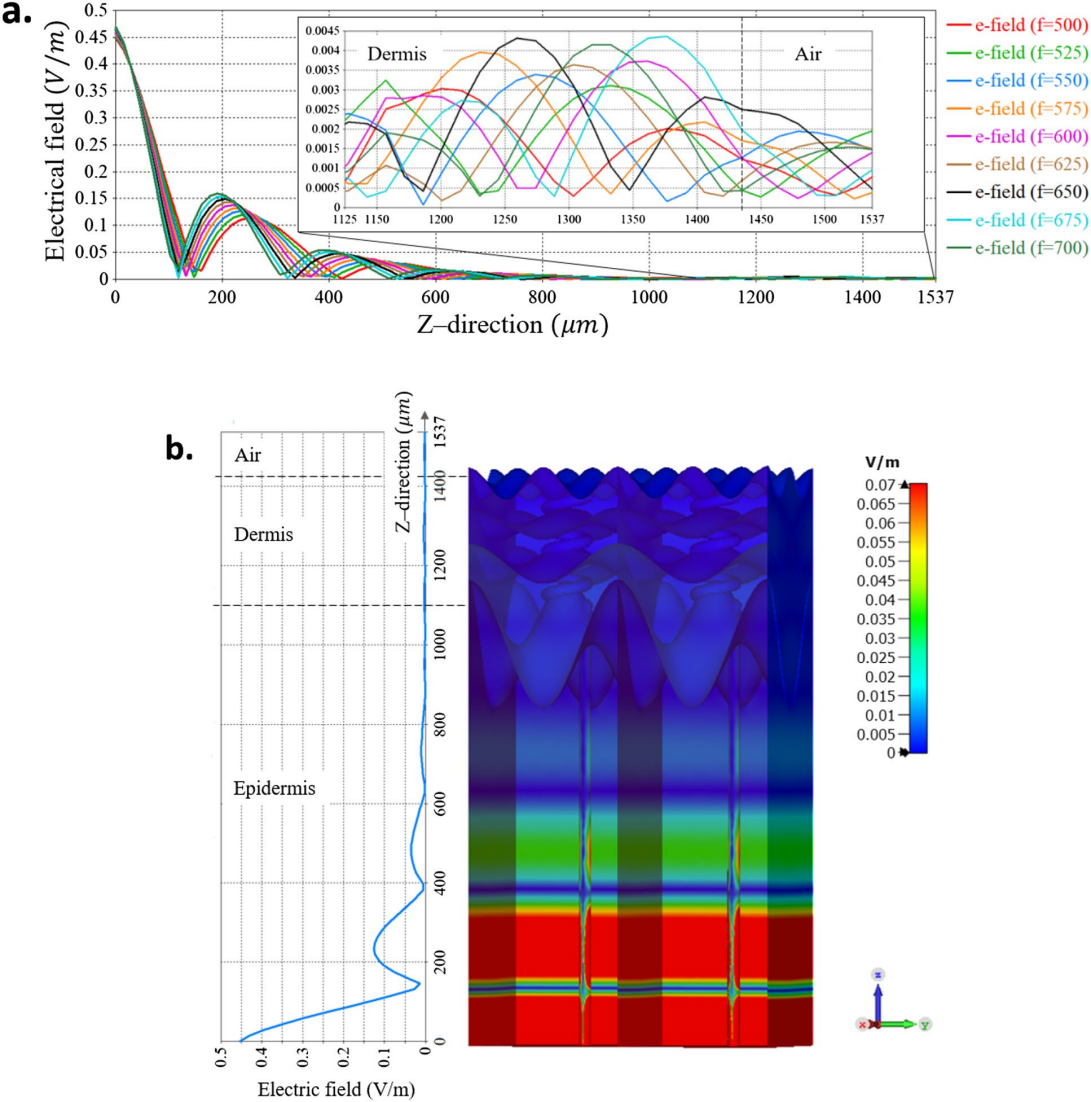
(**a**) The electrical field as function of distance in Z direction, i.e., throughout the skin model, for a fixed phase 145° and 9 different frequencies. (**b**) The electric field as function of the z-axis next to the 3D human skin model, for a perspective. This figure was generated using CST STUDIO SUITE v2021^30^, https://www.3ds.com/products-services/simulia/products/cst-studio-suite/.

Figure 9a shows the electric field averaged in the *xy*-plane, as function of *z*-axis, i.e., along the skin depth for the nine frequencies (500, 525, 550, 575, 600, 625, 650, 675 and 700 GHz). 0 μm is the bottom of the dermis layer, 1,125 μm is the location at which the dermal duct ends and the coiled section of the duct begins. At around 1,440 μm the skin interfaces with the outer surrounding. In the simulation this is air. In Fig. 9b the E-Field distribution, along with the averaged amplitude along the *z*-axis, is shown for f = 550 GHz.

**Figure 10:**
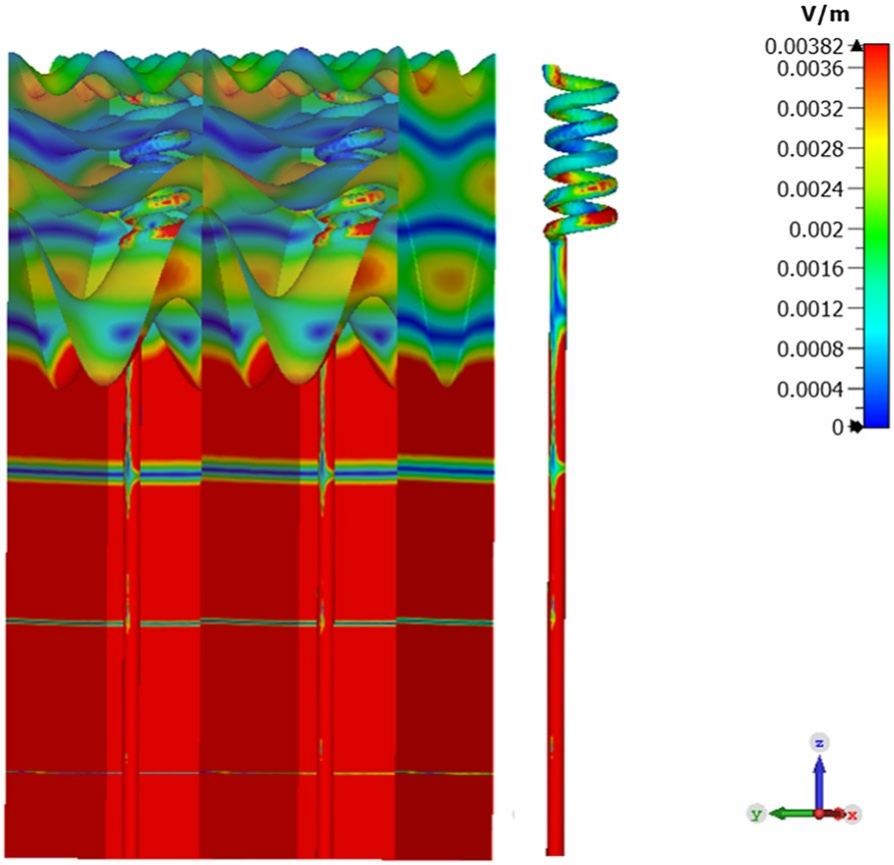
Electric field distribution in a fine resolution at 550 GHz. The sweat duct demonstrates performance of a conduit for the EM signal emitted from the core of the skin toward the skin surface This figure was generated using CST STUDIO SUITE v2021^30^, https://www.3ds.com/products-services/simulia/products/cst-studio-suite/.

As stated above and illustrated in Fig. 10 the decay of the field in the dermis is exponential and follows Beer’s law^31^, with an interference pattern superimposed upon it. Although greatly attenuated, this general picture is continued into the epidermis. The boundary between the skin and the air leads to a modification of the wave structure (see inset of Fig. 9a), as the wave propagates outward.

Figure 10 shows an enhanced image of the simulation whereby red indicates field strengths above 0.00382 V/m. The image reveals the fine structure of the field in the epidermis. One notes the effect of multiple reflections is to ‘compress’ the effective field envelope. Although the wavelength in the epidermis is approximately 320 microns, the thickness of the epidermis varies from 300 to 500 microns, depending on the position of the papillae. This leads to a more complex field profile and multiple interference peaks.

**Figure 11:**
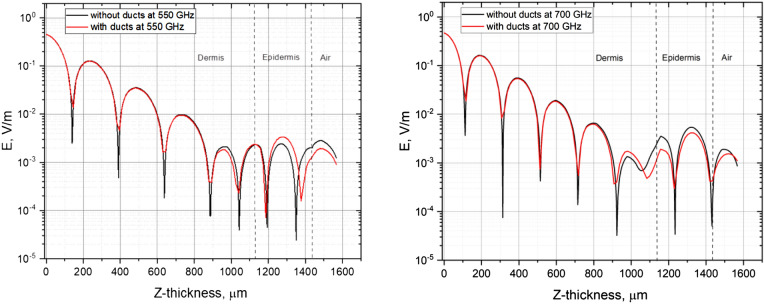
Log scale of the electric field as function of the skin depth. Left hand side is the simulation results for 550 GHz and the right hand side is the simulation results for 700 GHz. The continuous line is for the full model, including the sweat duct and the dashed line is the simulation results for a model without the sweat duct.

To investigate the role of the sweat duct in EM field structure, we have simulated the electrical field in two versions of the model; one that includes the sweat duct and one without the sweat duct. Figure 11 shows the electric field strength for two different frequencies, 550 and 700 GHz. It is clear the presence of the duct leads to a signal modulation by the absorption of energy in the epidermis (starting at approximately 1,125 microns and finishing at 1,440 microns) and deepens the interference pattern in the dermis.

Finally, we have compared the simulation results to the thermal radiation power measured from the human skin at 9 frequencies in the frequency range 500–700 GHz with a step of 25 GHz^5^. As described in the introduction, the brightness temperature has been measured for 3 subjects at these frequency points. Each subject was measured at each point 3 times^5^. The measurements were taken from the palm and so the skin can be considered as thick. The full description of these measurements and their results can be found in Ref. 5. In order to compare to the simulation results one first cast them in terms of an effective brightness temperature.

4 $$T_{B} \left( {f,T} \right) = \alpha \left( f \right) \cdot T$$

where $\alpha \left( f \right){ }$ is the grayness factor (emissivity) and $T$ is the physical temperature of the emitting object.

The grayness factor can be determined through the field energy losses in the skin during the waves propagation from the port (Z = 0 μm) to the surface (Z = 1,440 μm) by considering the intensity of the E-field at the skin surface, normalized by the input port field intensity $\left| {\frac{{E\left( {L,f} \right)}}{{E_{0} }}} \right|^{2}$, where the field value, $E\left( {L,{ }f} \right),$ is taken just above the skin surface at L = 1,537 μm. The value of the input field, $E_{o} ,$ can be estimated inside the skin from the definition of the simulation as $E_{o} \cong 1.3{ \times }10^{ - 3} \;{\text{V}}/{\text{m}}$ for a port power P = 5 nW, once the impedance mismatch at the port is taken into account. As a result, the intensity $I_{skin} \left( f \right)$ of the field in the skin is

5 $$I_{skin} \left( f \right) = 1 - \left| {\frac{{E\left( {L,f} \right)}}{{E_{0} }}} \right|^{2}$$

However, in the real radiometric experiment, the reflected wave passes into the region Z < 0 of body, and we have to assume that it would decay rapidly at the penetration depth. Thus, when comparing with experiment, we must assume that the energy of the field accumulated in the skin layer is completely absorbed and therefore Eq. (5) can be equated to the greyness factor, $\alpha \left( f \right) = I_{skin} \left( f \right)$.

**Figure 12:**
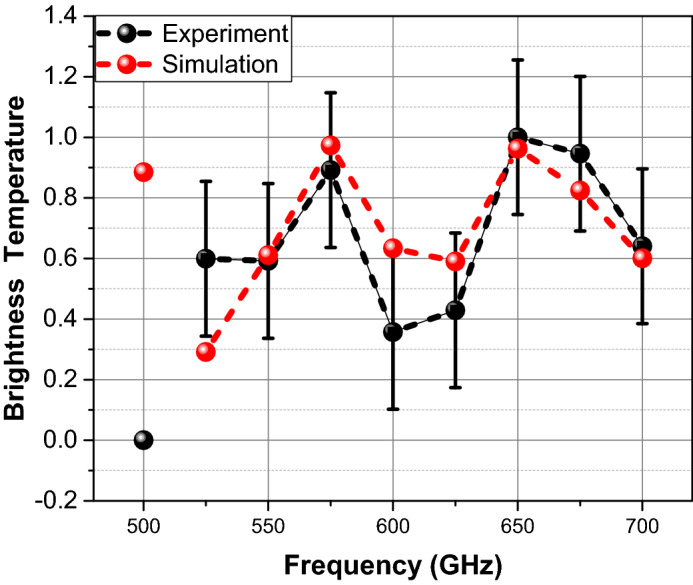
Comparison of the normalized skin brightness temperature calculated by CST simulations (Red) and by in vivo radiometric measurements of the skin thermal radiation (Black)^5^. The measurements accuracy is ± 1.7 degree. It can be seen that qualitatively the simulation and experiment results are having the same nature and follow the same trend.

Note that in simulations of wave propagation through the skin layer, its temperature $T$ was accounted for, while the measured brightness temperature in the radiometric experiment in vivo is proportional to the skin temperature $T$. Consequently, we compare the qualitative nature of both by their normalized values. Such comparison is shown in Fig. 12. The black solid spheres are the measurement, the red solid spheres are the simulation results. The qualitive behavior of the simulation result follows closely to that of the radiometric measurements.

## Conclusions

In this work, we have presented an EM model of the human skin, which takes into account the skin multi-layered structure and the sweat duct. For the first time, the dermal portion of the duct was considered. We conducted EM simulations in the frequency range of 500 GHz up to 700 GHz. The model reveals a complex interference structure of the E-field intensity in the skin. In the dermis, this pattern is overlain on a typical Beer’s law absorption. In the epidermis, multiple reflections from the skin surface and dermis/epidermis boundary lead to constructive interference. Furthermore, we trace the origin of the field the core blackbody radiation of the subject. As would be expected, the simulated greyness factor, as seen from the skin surface, replicates the measured radiometry intensities of our subjects^5^, we conclude that our simulation model is verified by experiment, lending confidence to the obtained interference patterns.

## Supplementary Information

Supplementary Information.
