# Supplementary material for: Emission from human skin in the sub THz frequency band
Source: Sci Rep. 2022 Mar 18;12:4720. doi: 10.1038/s41598-022-08432-5 (PMC8933490; doi:10.1038/s41598-022-08432-5)
Supplement: Supplementary file 1 — Supplementary Information. [file 41598_2022_8432_MOESM1_ESM.docx]

Supplementary

Using the EM simulations the electrical field was simulated for nine different frequencies in the range of 500 GHz up to 700 GHz with a constant frequency interval of 25 GHz (500, 525, 550, 575, 600, 625, 650, 675 and 700 GHz). For each frequency, the field distribution was recorded for the same arbitrary fixed phase value of$145^{\circ}$. Figure S1 demonstrate the results all nine frequencies, which were for an absolute distribution. All simulations were made for a sweat duct conductivity of $1,000 S/m$ and a plane wave source of average$5 nW$. Figure S1 is the complete data of figure 7 in the main text.


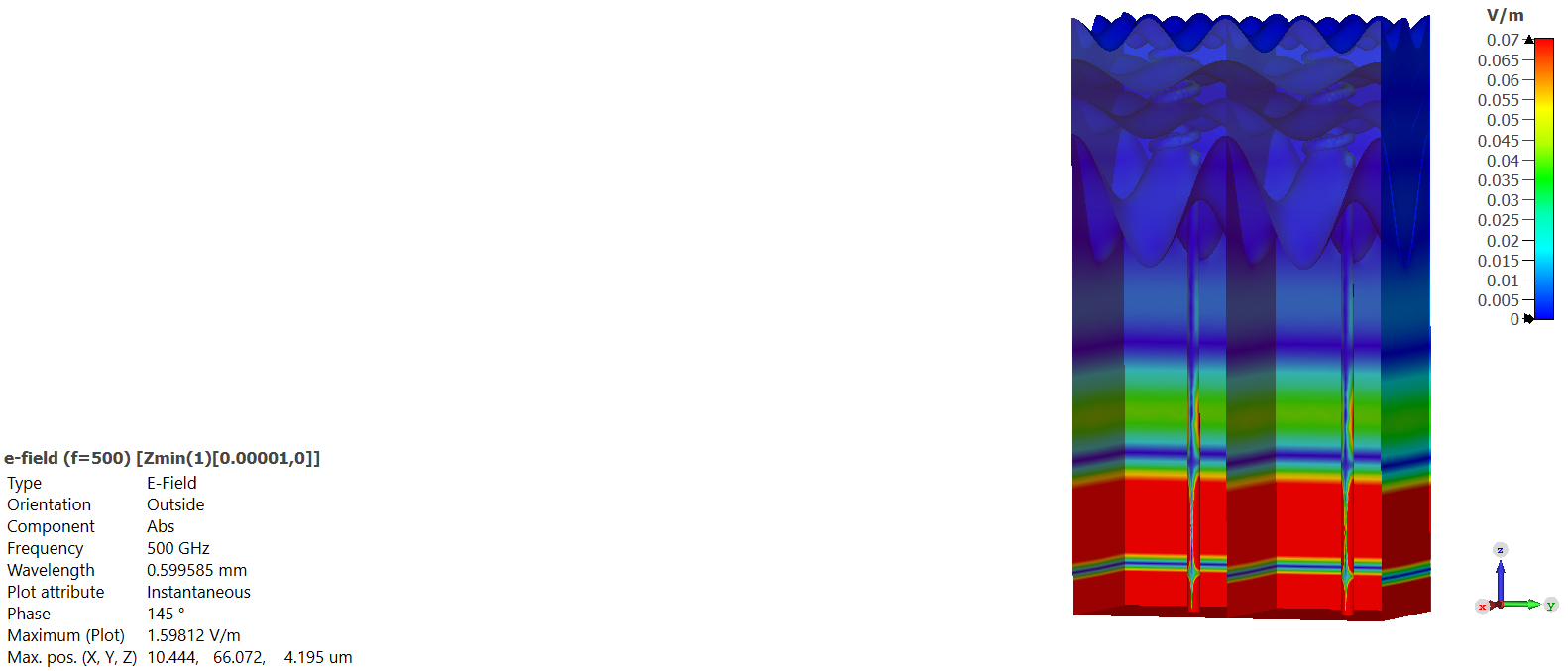


**a***.*

**b***.*


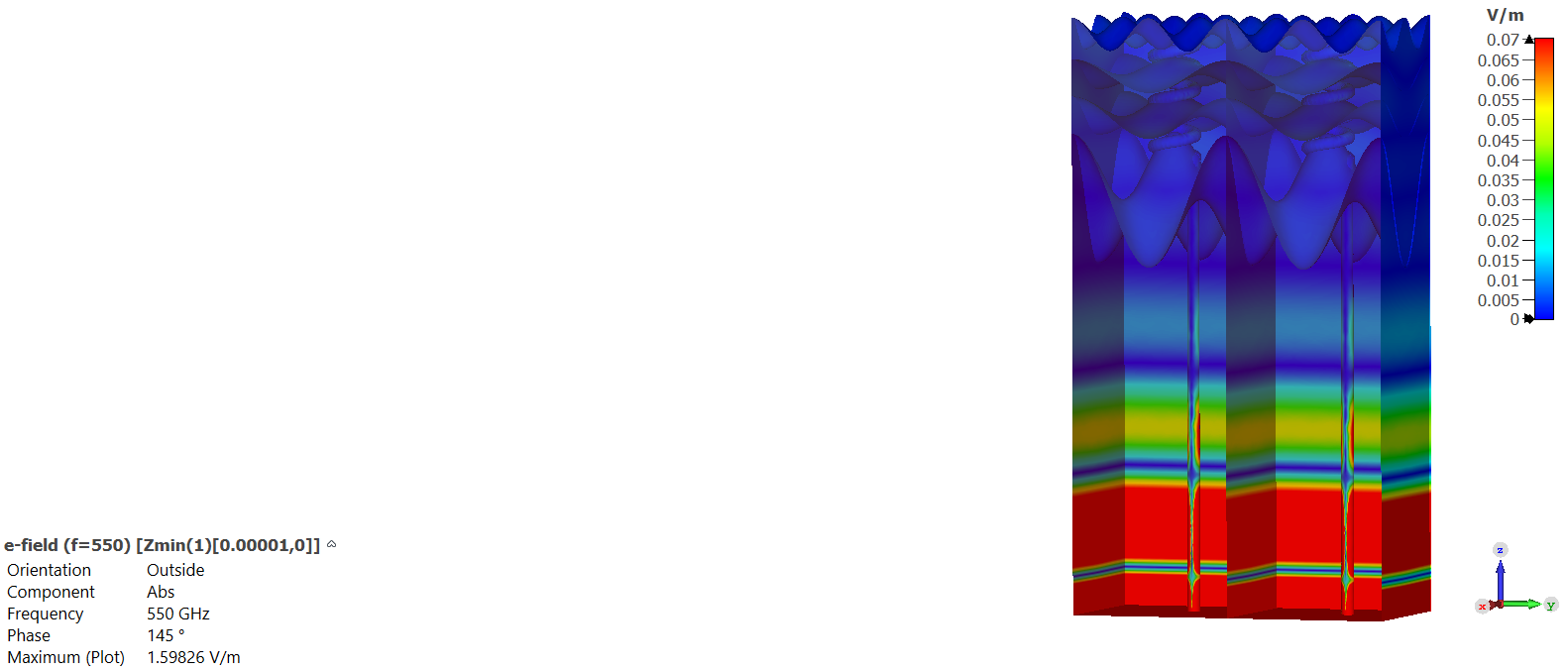


**c.**


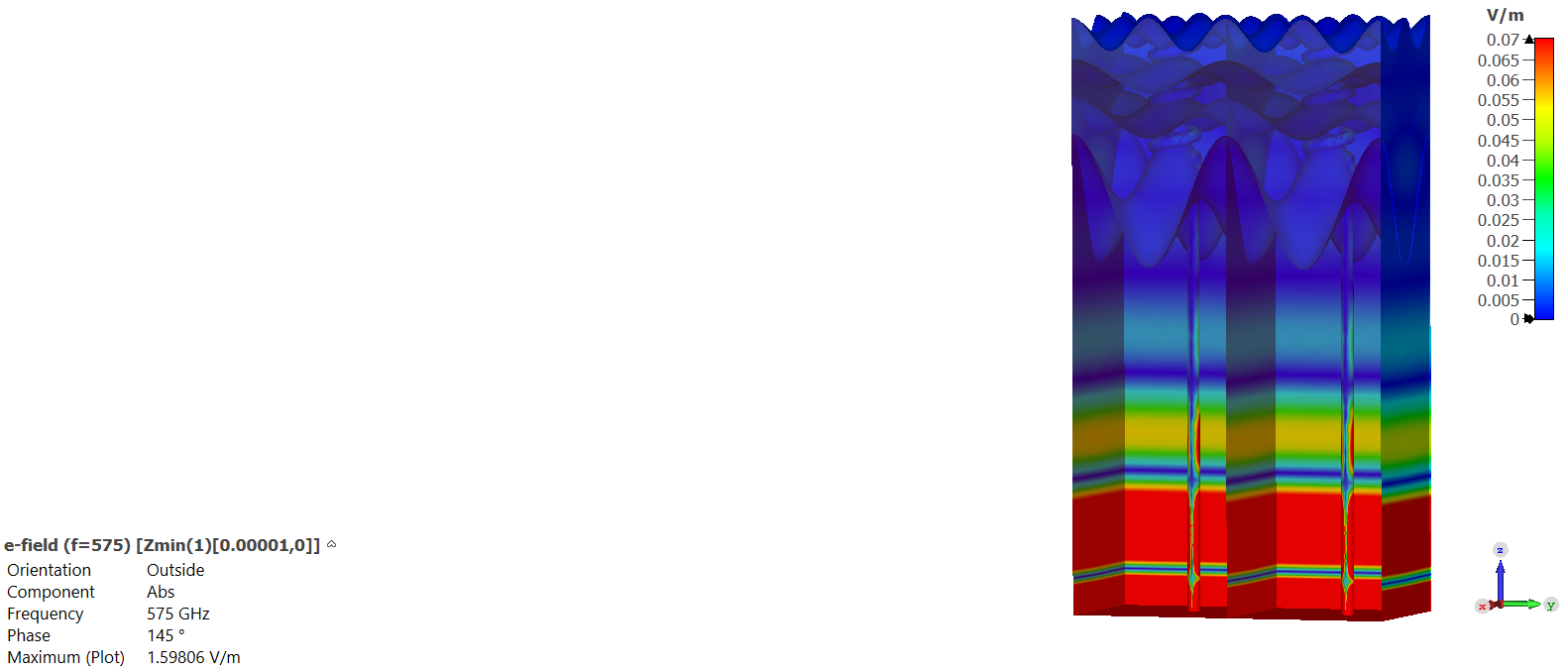


**d***.*


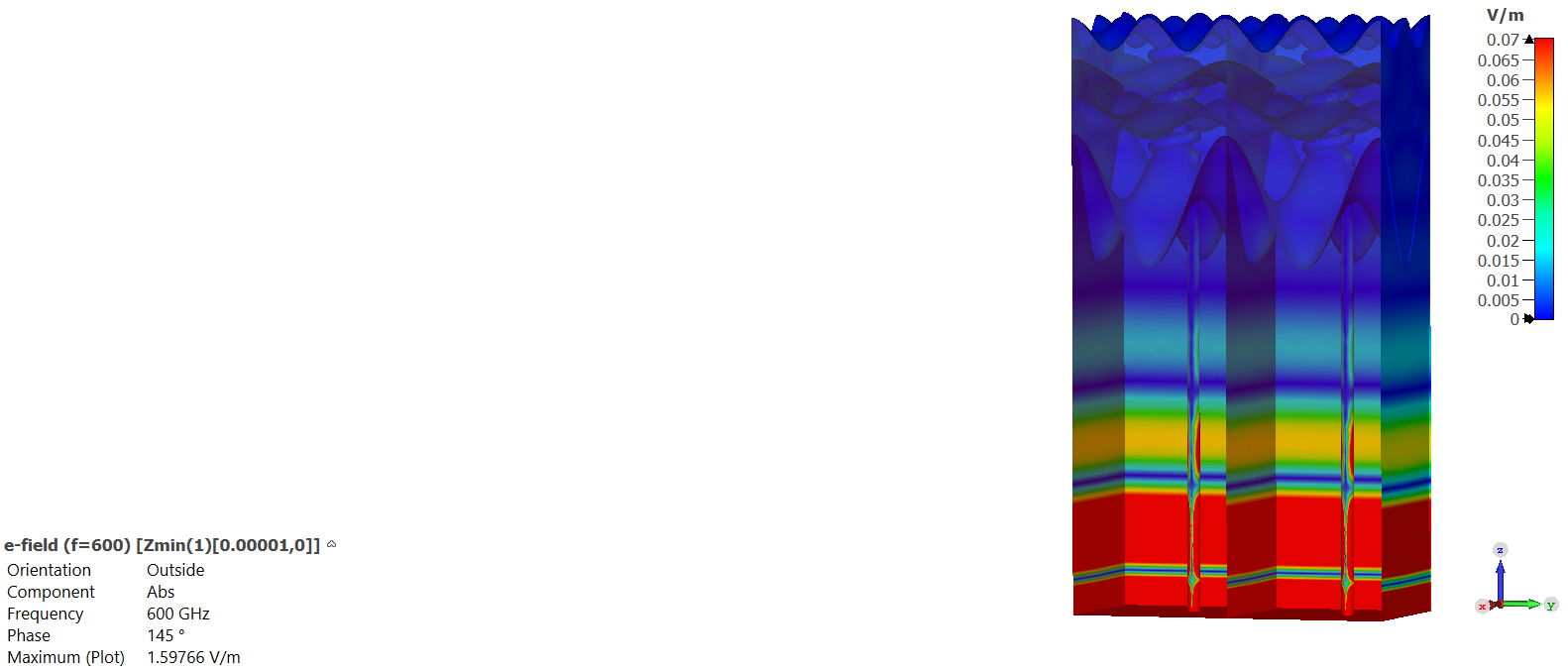


**e***.*


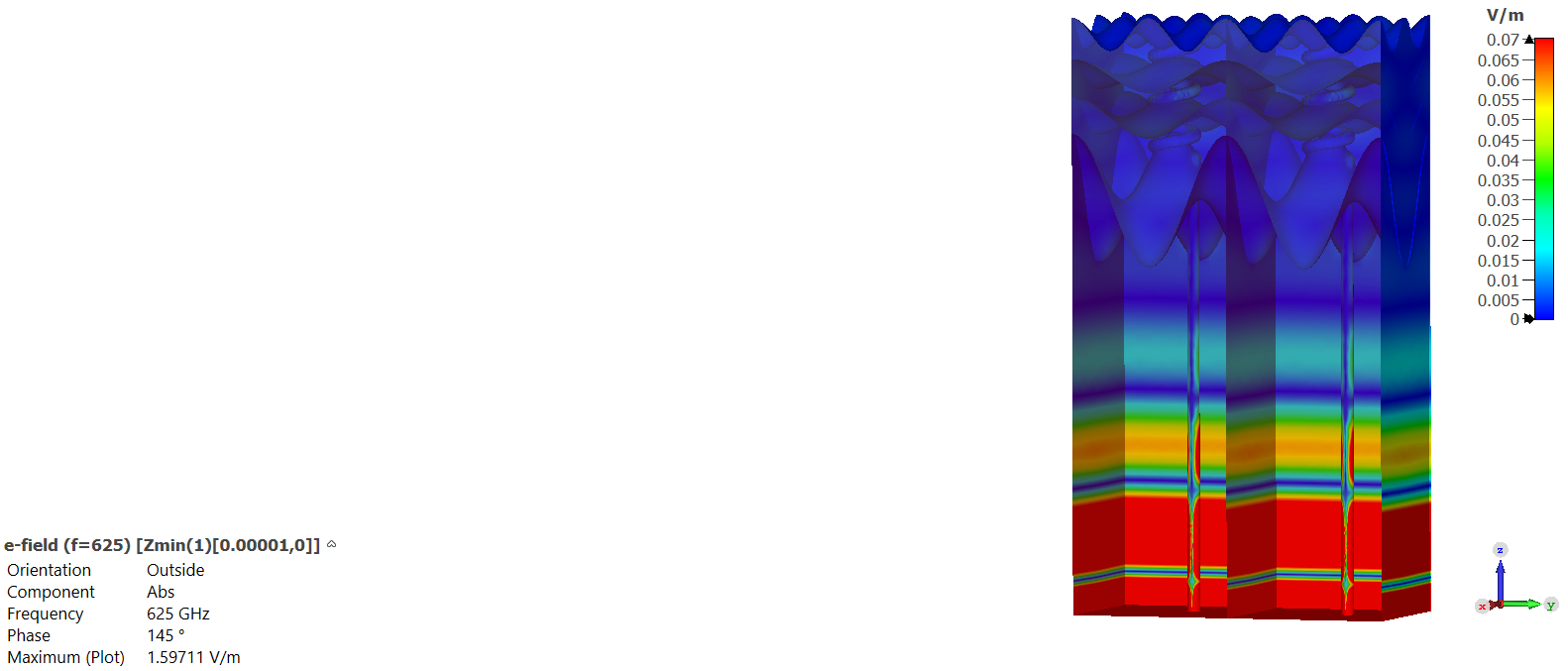


**f***.*


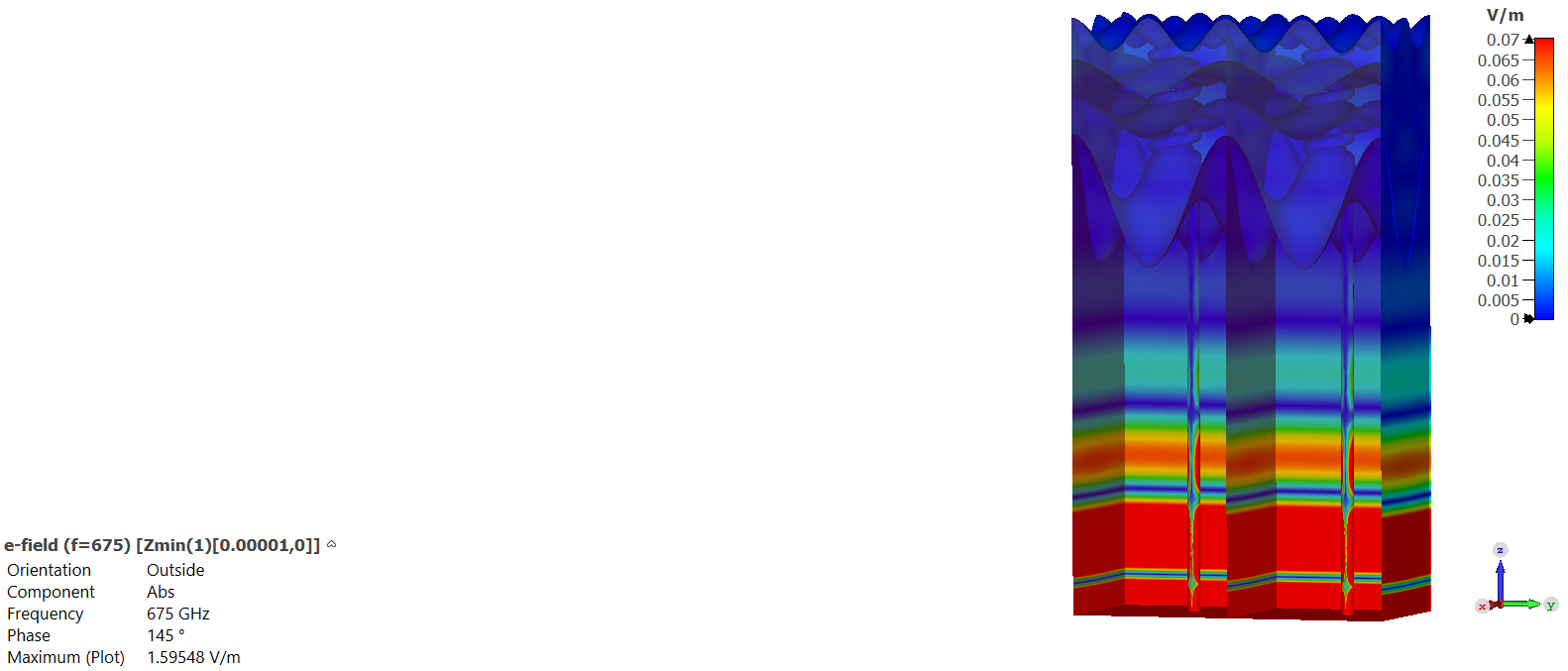


**h***.*


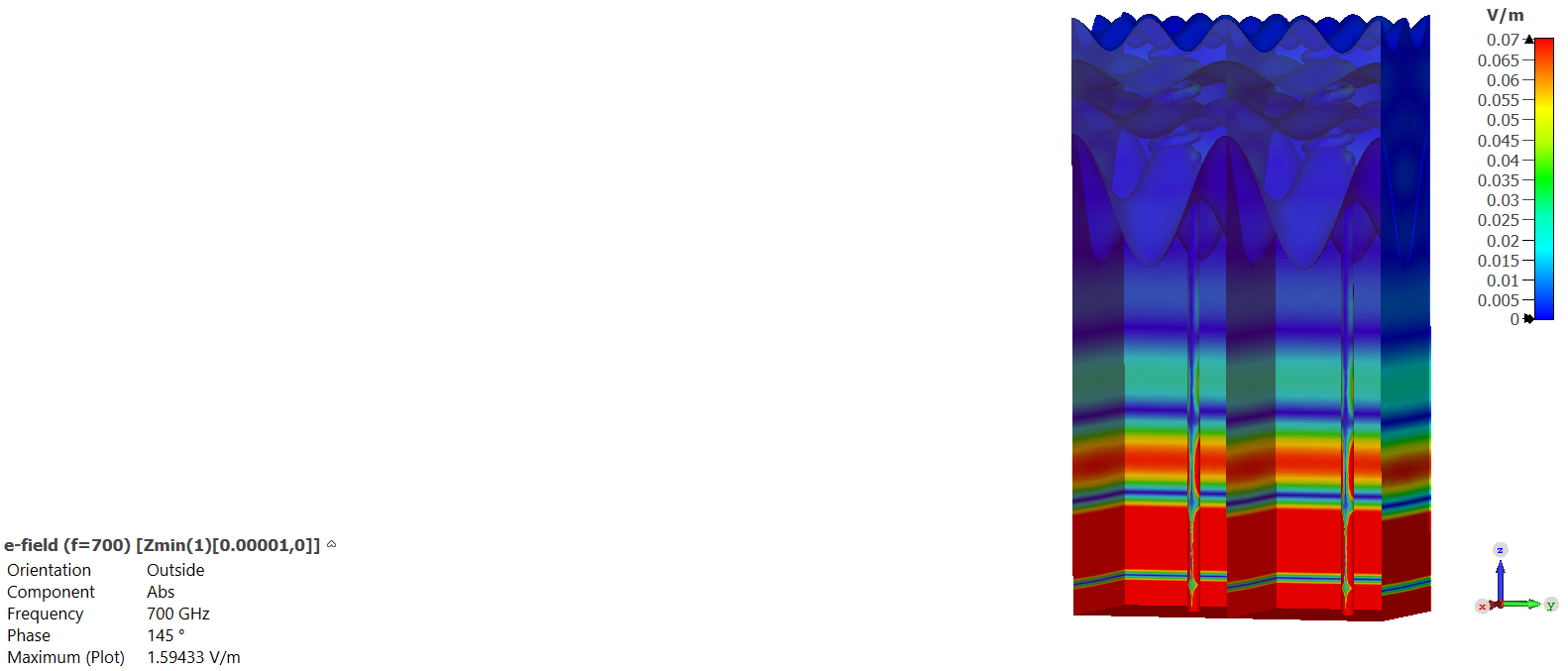


**i.**


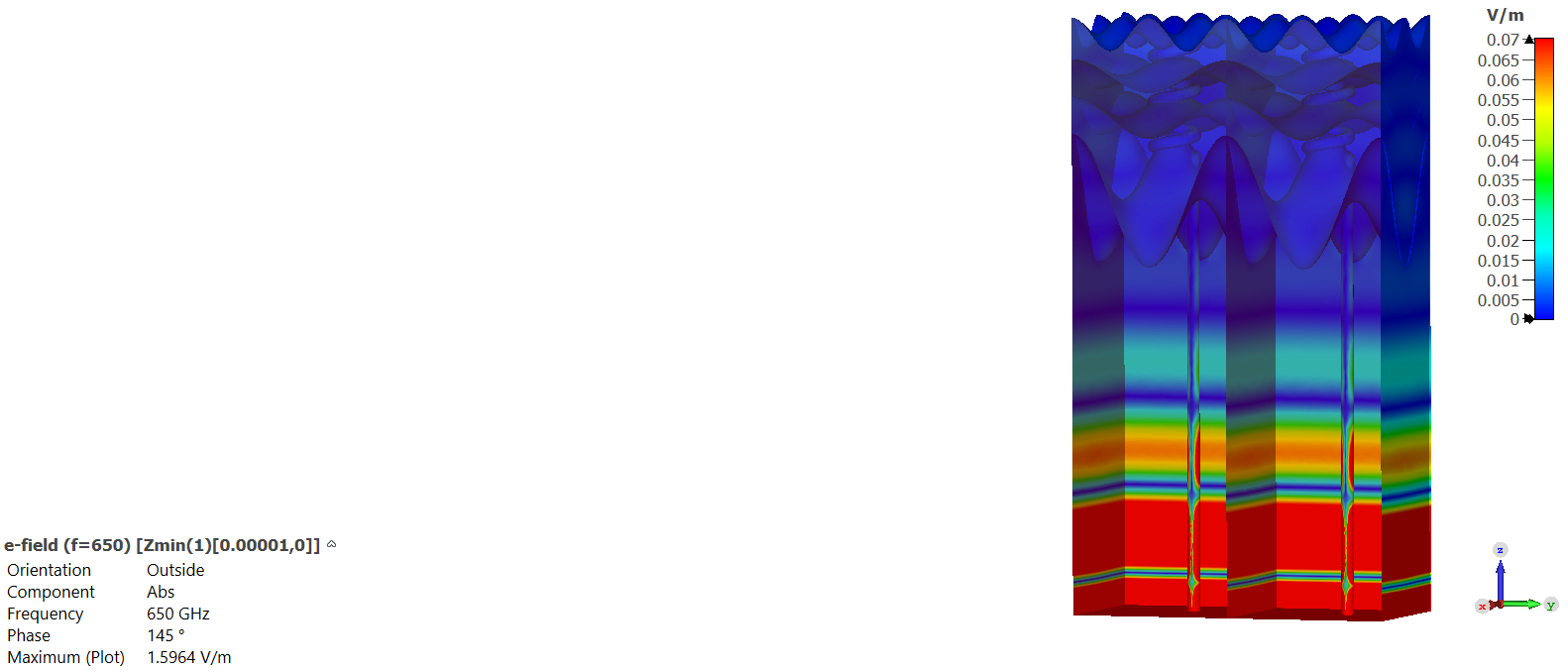


**g***.*

Figure S1. Electrical field distribution simulated for nine different frequencies in the range of 500 GHz up to 700 GHz with a constant frequency interval of 25 GHz. (**a**. 500 GHz, **b**. 525 GHz, **c**. 550 GHz, **d**. 575 GHz, **e**. 600 GHz, **f**. 625 GHz, **g**. 650 GHz, **h.** 675 GHz, and **i**. 700 GHz). The duct conductivity is 1000 S/m and power average of 5 nW. The phase for all frequencies was fixed to $145^{\circ}.$ the appearance of a complex wave structure from frame d onwards.

In figure S2, the simulation is for a single frequency – 550 GHz – but with different initial phases ($0^{\circ}, 45^{\circ}, 90^{\circ}, 135^{\circ}, 180^{\circ}, 225^{\circ}, 270^{\circ}, 315^{\circ} and 360^{\circ})$, representing the passage of the plain wave through the strata. Figure S2 is the complete data of figure 8 in the main text.


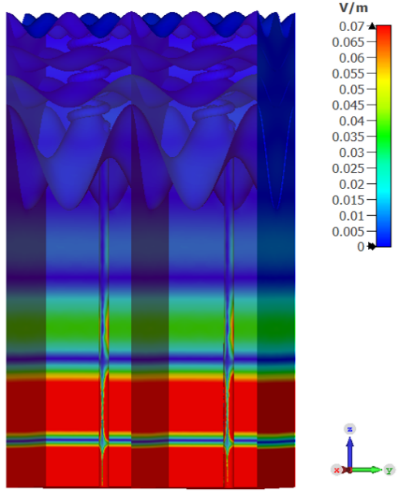


**a.**

**b.**


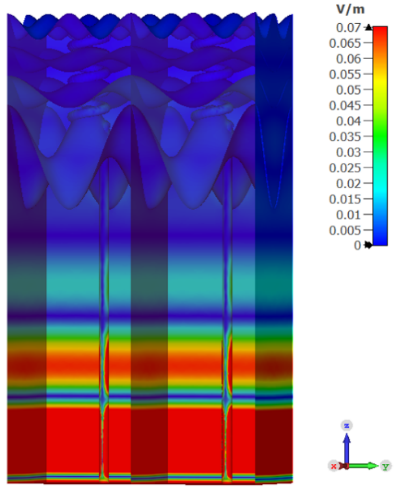


**c.**


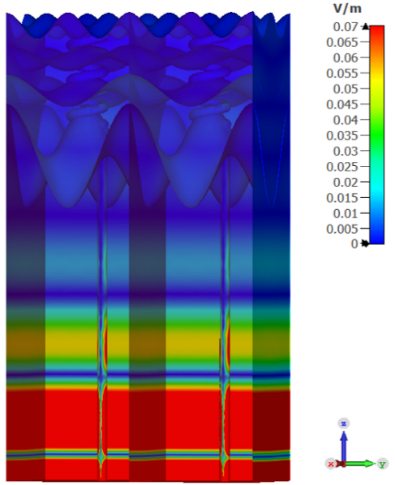


**d.**


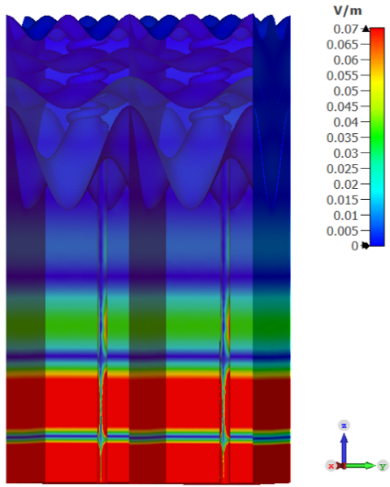


**e***.*


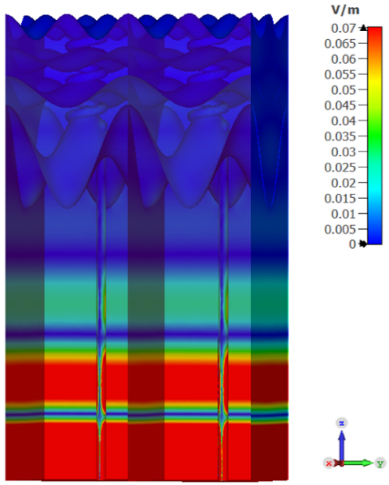


**f***.*


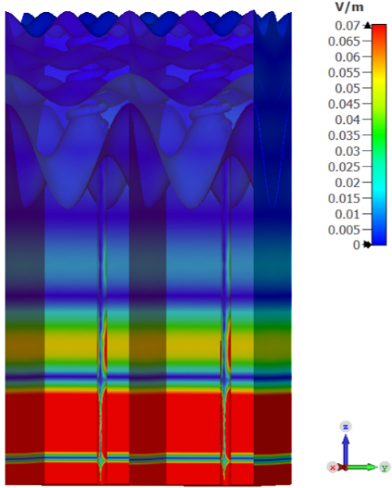


**h***.*


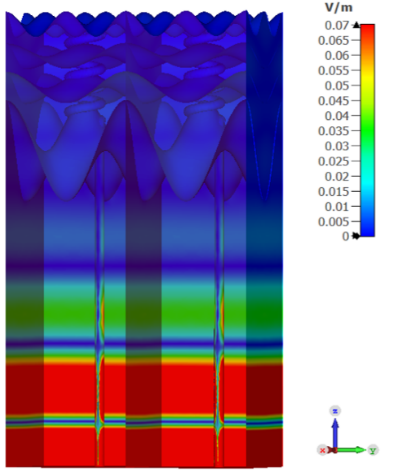


**i.**


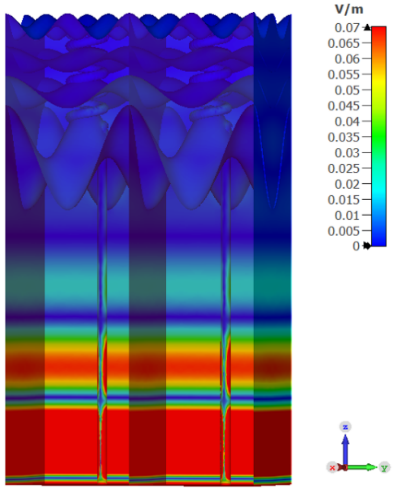


**g***.*

Figure S2. Fixed frequency of 550 GHz and different phases (**a**.$0^{\circ}$, **b**. $45^{\circ}$, **c**. 90$^{\circ}$, **d**.135$^{\circ}$, **e**. $180^{\circ}$, **f**. 225$^{\circ}$, **g**. $270^{\circ}$, **h.** 315$^{\circ}$, and **i**$360^{\circ}$). In the scale-bar, all values above 0.07 V/m are marked in Red color.
